# OmicsEV: a tool for comprehensive quality evaluation of omics data tables

**DOI:** 10.1093/bioinformatics/btac698

**Published:** 2022-10-22

**Authors:** Bo Wen, Eric J Jaehnig, Bing Zhang

**Affiliations:** Lester and Sue Smith Breast Center, Baylor College of Medicine, Houston, TX 77030, USA; Department of Molecular and Human Genetics, Baylor College of Medicine, Houston, TX 77030, USA; Lester and Sue Smith Breast Center, Baylor College of Medicine, Houston, TX 77030, USA; Department of Molecular and Human Genetics, Baylor College of Medicine, Houston, TX 77030, USA; Lester and Sue Smith Breast Center, Baylor College of Medicine, Houston, TX 77030, USA; Department of Molecular and Human Genetics, Baylor College of Medicine, Houston, TX 77030, USA

## Abstract

**Summary:**

RNA-Seq and mass spectrometry-based studies generate omics data tables with measurements for tens of thousands of genes across all samples in a study. The success of a study relies on the quality of these data tables, which is determined by both experimental data generation and computational methods used to process raw experimental data into quantitative data tables. We present OmicsEV, an R package for the quality evaluation of omics data tables. For each data table, OmicsEV uses a series of methods to evaluate data depth, data normalization, batch effect, biological signal, platform reproducibility and multi-omics concordance, producing comprehensive visual and quantitative evaluation results that help assess the data quality of individual data tables and facilitate the identification of the optimal data processing method and parameters for the omics study under investigation.

**Availability and implementation:**

The source code and the user manual of OmicsEV are available at https://github.com/bzhanglab/OmicsEV, and the source code is released under the GPL-3 license.

## 1 Introduction

RNA-Seq and mass spectrometry (MS)-based proteomics provide global measurements of the abundance of genes and their protein products in samples of interest. These measurements are usually stored in quantitative data tables for downstream analysis. The success of an omics study is largely determined by the quality of these tables, which in turn depends on the quality of both experimental data generation and computational methods used to process raw experimental data into quantitative data tables. Omics data processing involves multiple steps, and many algorithms, software, and workflows have been developed for these essential tasks.

Optimal selection of tools, algorithms and parameters is critical to ensure reliable and accurate estimation of gene or protein quantification (Conesa *et al.*, 2016). Many studies have been performed to evaluate different methods for processing RNA-Seq or proteomics data (Cole *et al.*, 2019; Corchete *et al.*, 2020; Frohlich *et al.*, 2022; Valikangas *et al.*, 2018). Many of these studies focus on a specific aspect of data processing such as normalization or missing value imputation. Moreover, these studies are typically based on a few benchmarking datasets. Due to the complexity of omics data and its processing, a single evaluation study does not cover all aspects of the data processing and may not generalize to other datasets with different complexities. In addition, as new methods are being continuously developed, new evaluation studies are required. It remains a challenge to pick optimal methods and parameter settings for processing a specific omics dataset of interest.

Here, we propose OmicsEV, an R package that provides multiple quality evaluation methods for RNA-seq and proteomics data tables. These metrics can be used to evaluate the quality of a given RNA or protein data table and to guide the selection of optimal methods or parameter settings for processing a specific dataset.

## 2 Features and implementation

**Fig. 1.**
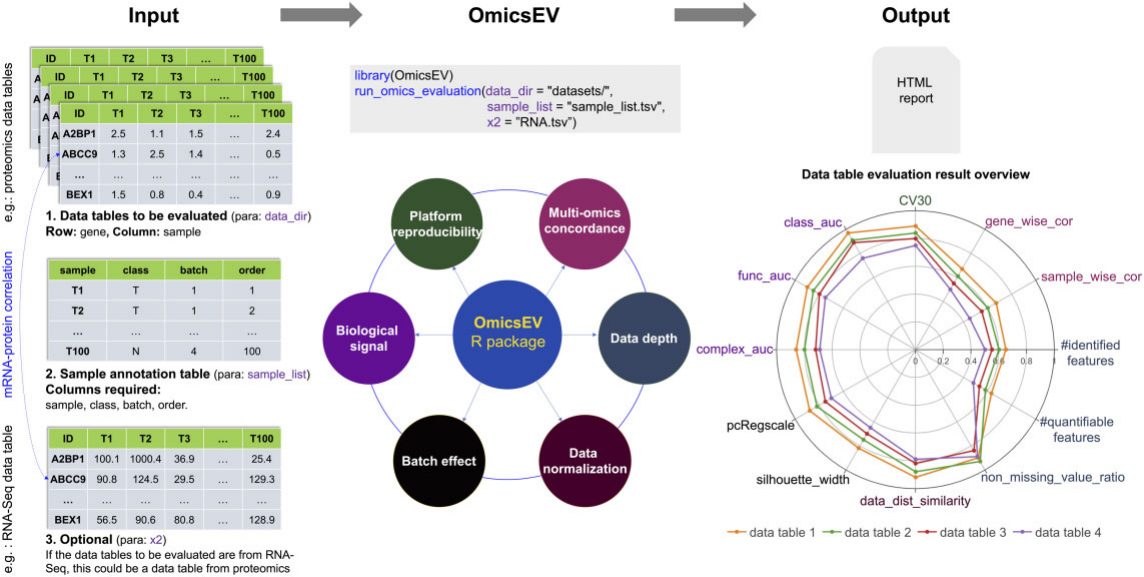
An overview of OmicsEV

OmicsEV is implemented as an R package, and a docker is also available. OmicsEV takes a folder that contains omics data tables to be evaluated together with a sample annotation file as input and evaluates the data quality of each data table using a series of methods, which can be classified into six groups focusing on different aspects (Fig. 1). Group 1 methods assess data depth. The numbers of identified and quantifiable features (i.e. genes or proteins) in each data table are summarized in a table. The overlap of features across different data tables is visualized in an UpSet plot. A scatter plot is used to visualize the number of features identified in each sample. Moreover, the missing value distribution plots provide an overview of the missing data frequency in each data table. Group 2 methods evaluate data normalization by visualizing feature abundance distribution in each sample using boxplots and density plots and by computing pair-wise distribution similarities based on the receiver operating characteristic curve (AUROC) analysis. Group 3 methods evaluate the potential batch effect. Batch effect is quantified using silhouette width and principal component regression analysis. These quantifications are complemented by the visualization of principal component analysis (PCA) plots and correlation heatmaps. Group 4 methods assess the strength of the biological signal in a data table using four complementary methods. First, correlation analysis based on protein complexes from the CORUM database is performed. For each data table, the same number of intra-complex feature pairs and inter-complex pairs are randomly generated, and the Pearson correlation coefficient is calculated for each pair. The correlations of inter-complex pairs should be close to zero, and higher overall correlations for intra-complex pairs are indicative of stronger biological signal and better data quality. Second, gene function prediction is performed using co-expression network analysis (Wang *et al.*, 2017). Each data table is used to build a co-expression network of features. For a selected network and a selected KEGG pathway, features annotated to the pathway and included in the network are defined as a positive feature set, and other features in the network constitute the negative set for the pathway. The random walk with restart algorithm is then used to assess prediction performance for each pathway and network through cross-validation (Wang *et al.*, 2017). Better performance as quantified by higher AUROC scores indicates stronger biological signal and better data quality. Third, for a sample classification specified in the input file, e.g. tumor versus normal, machine learning models are constructed based on each data table, and the prediction performance evaluated through cross-validation is compared across data tables. Higher AUROC scores correspond to stronger biological signal and better data quality. Finally, unsupervised clustering is used to visually assess whether sample grouping is associated with the sample feature specified in the input file, indicating good data quality, or experimental batches, indicating batch effect. Group 5 methods evaluate platform reproducibility when replicated quality control (QC) samples are included in the study. The coefficient of variation (CV) for each feature is computed for replicated QC samples in each data table. CV distributions are plotted, and the percentage of features with CV less than 30% is used as a quantitative metric for platform reproducibility assessment. Group 6 methods focus on multi-omics concordance. When a paired mRNA data table is available for proteomics data tables, mRNA-protein correlation metrics for gene-wise and sample-wise correlations are generated. Higher overall correlation is indicative of the better quality of a proteomics data table. When evaluating RNA-Seq data tables, a paired proteomics data table can be used similarly if available. Together, these six groups of methods produce comprehensive visual and quantitative evaluation results that help evaluate the quality of a given data table and facilitate the identification of the optimal data processing method and parameters for the omics study under investigation. All these analyses are wrapped into a single function for easy application. All evaluation results are included in an HTML report, preceded by a brief introduction section summarizing the data tables under evaluation and associated sample information and by an overview section with all quantitative results summarized in a table and visualized in a radar plot (Fig. 1).

## 3 Applications

Earlier versions of OmicsEV have been used in multiple proteogenomic studies on hepatocellular carcinoma (Gao *et al.*, 2019), endometrial carcinoma (Dou *et al.*, 2020), breast cancer (Satpathy *et al.*, 2020), head and neck squamous cell carcinoma (Huang *et al.*, 2021) and pancreatic ductal adenocarcinoma (Cao *et al.*, 2021), respectively. Three examples on the application of the current version of OmicsEV to evaluate RNA-Seq data tables generated using different normalization methods, proteomics data tables generated using different pipelines and a single RNA-Seq data table, respectively, are available at https://github.com/bzhanglab/OmicsEV. We expect OmicsEV will have broad applications in omics studies to assess data quality and to select and optimize data processing methods including tools, algorithms and parameter settings.

## Funding

This work was supported by grants [U24CA210954 and U24CA271076] from the National Cancer Institute Clinical Proteomic Tumor Analysis Consortium (CPTAC), by grant CPRIT [RR160027] from the Cancer Prevention & Research Institutes of Texas, by funding from the McNair Medical Institute at The Robert and Janice McNair Foundation. B.Z. is a CPRIT Scholar in Cancer Research and a McNair scholar.

*Conflict of Interest*: none declared.
